# Dynamic neuronal regulatory network during the progression of Alzheimer’s disease suggests an adaptive survival strategy

**DOI:** 10.1186/1750-1326-7-S1-O3

**Published:** 2012-02-07

**Authors:** Jiya Sun, Xuemei Feng, Dapeng Liang, Yong Duan, Hongxing Lei

**Affiliations:** 1 Beijing Institute of Genomics, Chinese Academy of Sciences, Beijing, 100029, China; 2 Graduate University, Chinese Academy of Sciences, Beijing, 100049, China; 3 UC Davis Genome Center and Department of Applied Science, One Shields Avenue, Davis, CA 95616, USA; 4 College of Physics, Huazhong University of Science and Technology, Wuhan, 430074, China

## 

Alzheimer’s disease (AD) is the most common neurodegenerative disease. In this work, comprehensive analyses on transcriptome studies of human postmortem brain tissues from AD patients revealed stepwise breakdown of the cellular machinery during the progression of AD at semi-quantitative level. At the early stage of AD, the accumulation of A-beta oligomers and amyloid plaques leads to the down-regulation of biosynthesis and energy metabolism, likely a response to the reduced level of nutrient and oxygen supply. At the intermediate stage, the progression of the disease leads to enhanced signal transduction, also likely an adjustment to the deteriorating environment. The late stage is characterized by the elevated apoptosis due to the excessive regulatory and repair burden. Interestingly, the elevated apoptosis at the late stage is accompanied by elevated anti-apoptotic signals, suggesting an adaptive survival strategy even at the late stage of AD. These findings can serve as theoretical basis for pharmaceutical intervention of Alzheimer’s disease.

